# Neutrophils in the Bone Marrow Express DEC205, Guiding Their Migration to Inflamed Tissues

**DOI:** 10.1111/imm.13958

**Published:** 2025-06-04

**Authors:** Shiying Jin, Xuan Lei, Sonali Singh, Alexander Enk, Karsten Mahnke

**Affiliations:** ^1^ Department of Dermatology University Hospital Heidelberg Heidelberg Germany

**Keywords:** adhesion molecules, cell surface molecules, cell trafficking, inflammation, neutrophils

## Abstract

The surface molecule DEC205 (CD205) is well‐characterised in murine dendritic cells (DCs) as an antigen uptake receptor. However, recent studies have also identified its expression in other leukocytes, including B cells and neutrophils. In B cells, DEC205 functions as an endocytic receptor, similar to its role in DCs. Since neutrophils are not professional antigen‐presenting cells, we sought to investigate the functions of DEC205 beyond endocytosis. Analysis of cell surface expression of DEC205 in neutrophils at different maturation stages and anatomical locations revealed its downregulation upon in vitro activation and during tissue infiltration, such as in skin inflammation and thioglycolate‐induced peritonitis. In DEC205‐deficient (DEC205^−/−^) mice, neutrophils exhibited reduced migration in Boyden chamber assays and impaired accumulation in the peritoneum, along with decreased adhesion to extracellular matrix proteins. These findings suggest that, beyond its established role as an antigen uptake receptor in antigen‐presenting cells, DEC205 may serve as a marker for early‐stage neutrophils with the capacity to migrate and infiltrate tissues.

## Graphical abstract

In bone marrow, immature neutrophils express high levels of DEC205, necessary to mediate migration through endothelial cell layers in vitro and in vivo. During maturation and migration to peripheral tissues, expression of DEC205 is downregulated. Thus, expression of DEC205 inversely correlates with the maturation of neutrophils and is involved in guiding them to inflamed tissues.

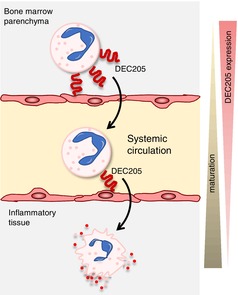

**Abbreviations**
7‐AAD: 7‐Aminoactinomycin D
BM: bone marrow
CHS: contact hypersensitivity
CRDs: carbohydrate recognition domains
DC: dendritic cell
DEC205^−/−^
: DEC205‐deficient
ECM: extracellular matrix
ELISA: enzyme‐linked immunosorbent assay
fMLP: *N*‐formyl‐methionyl‐leucyl‐phenylalanine
FNII: fibronectin‐II domain
G‐CSF: granulocyte‐colony stimulating factor
LN: lymph node
MMR: macrophage mannose receptor
PEC: peritoneal exudate cells
PMA: phorbol 12‐myristate 13‐acetate
RFI: relative fluorescence intensity
TNCB: 2,4,6‐trinitro‐1‐chlorobenzene
WT: wildtype

## 1 Introduction

The surface molecule DEC205 has extensively been characterised in murine DCs and serves as an antigen uptake receptor in dendritic [1, 2, 3, 4, 5] and Langerhans cells [6, 7]. However, recent investigations reassessed the expression pattern of DEC205 by leukocyte subsets other than DCs [8, 9]. These investigations revealed the expression of DEC205 by B cells and neutrophilic granulocytes (neutrophils). As for B cells, which are antigen‐presenting cells similar to DCs, the function of DEC205 as an endocytic receptor has been established [10, 11].

In contrast to B cells and DCs, neutrophils have only a limited capacity to express major histocompatibility complex class II molecules and harbour not a complete antigen processing machinery to effectively present antigens to T cells [12, 13]. This makes it highly unlikely that DEC205 functions as an antigen receptor in neutrophils. Moreover, not many data are available on the regulation of DEC205 expression by neutrophils. Most of the investigations of DEC205‐expressing neutrophils rely on the isolation of neutrophils from the bone marrow (BM) [14]. These neutrophils are not fully matured, as they have to migrate to peripheral tissues where they are involved in the first line of defence against pathogens. As neutrophils act in a suicide mission by NETosis and reactive oxygen species production, they most likely die in the periphery [15]. Thus, an antigen presenting function, which would require them to return to regional lymph nodes (LNs) for T cell activation, is rather unlikely.

When assessing the enigmatic function(s) of DEC205 in neutrophils by analysing the expression pattern in different maturation stages and tissue localizations in mice, we found that DEC205 expression by BM‐derived neutrophils is downregulated by activation in vitro. Likewise, tissue‐invading neutrophils lose DEC205 expression in skin inflammations such as contact hypersensitivity (CHS) reaction, and become DEC205^−^ in the peritoneum during thioglycolate‐induced peritonitis. When the migratory capacity of neutrophils in DEC205^−/−^ animals was tested, we found reduced migration of these cells in Boyden chamber experiments and a severely impaired accumulation in the peritoneal cavity upon induction of peritonitis by thioglycolate, as compared to wildtype (WT) controls. This lack of migration was accompanied by a reduced adhesion of DEC205^−/−^ neutrophils to extracellular matrix (ECM) proteins. Thus, these data indicate that in neutrophils DEC205 expression marks immature neutrophils in the blood and the BM, and that DEC205 acts as a molecular marker for immature neutrophils capable of migrating to peripheral tissues. Once invaded into tissues, neutrophils eventually terminally mature and DEC205 expression is downregulated as it is no longer necessary for targeting and movement to peripheral organs.

## 2 Materials and Methods

### 2.1 Mice

C57BL/6 WT 5‐ to 7‐week‐old mice used in this study were purchased from the Janvier Labs (Le Genest‐Saint‐Isle, France). DEC205^−/−^ 5‐ to 7‐week‐old mice (B6.129P‐Ly75tm1Mnz/J) were purchased from the Jackson Laboratory (Bar Harbour ME, USA). LysM‐eGFP mice were generated by Dr. Graf [16]. All mice were bred and housed in specific pathogen‐free conditions at the animal facility of Heidelberg University. All animal experiments were conducted in accordance with the guidelines for animal welfare established by the state of Baden–Württemberg (G53/15 and G2/24) and were approved by the relevant authorities.

### 2.2 CHS Model

WT and DEC205^−/−^ mice were sensitised by 15 μL of 1% 2,4,6‐trinitro‐1‐chlorobenzene (TNCB) (Sigma, Deisenhofen, Germany) dissolved in acetone/olive oil (4:1) on the shaved abdomen. On Day 5, challenge was performed with 10 μL of 0.5% TNCB on both sides of one ear. After 24 h, mice were sacrificed to collect several organs. For intravenous injection experiments, half of the recipient mice were sensitised by 10 μL of 1% TNCB on both sides of one ear, while the other half were treated only with acetone/olive oil (4:1) in the same way. Mice were sacrificed after 24 h.

### 2.3 Acute Peritonitis Model

4% thioglycolate medium was prepared according to standard protocols [17, 18] and stored dark at room temperature to age for 4 weeks. The sterile peritonitis was induced by the intraperitoneal injection of 1.0 mL sterile 4% thioglycolate medium. Control mice were injected intraperitoneally with 1.0 mL sterile PBS. Two or four hours later, mice were sacrificed to collect BM, blood and peritoneal exudate cells (PECs).

### 2.4 Single‐Cell Suspension Preparation

#### 2.4.1 BM, Blood, LNs, Spleen and PECs

For BM cells, the femurs and tibias of mice were flushed out, and then the cells were filtered, followed by lysing in 10 mL of ACK lysis buffer (Sigma, Schnelldorf, Germany) for 3 min. The blood samples were lysed in 20 and 10 mL of ACK lysis buffer for 3 min twice. LNs and spleen were mashed gently and then filtered. Splenocyte single‐cell suspensions were lysed in 3 mL of ACK lysis buffer for 3 min. PECs were collected from thioglycollate‐treated or PBS‐treated mice by washing the peritoneal cavity with 5 mL of ice‐cold PBS [19].

#### 2.4.2 Ears and Lungs

Mice ears were split into dorsal and ventral halves and then incubated at 37°C for 30 min in 15 mL of trypsin lysis buffer [20]. The digestion was terminated by adding 10 mL EC‐stop buffer (PBS, 20% FCS, 0.1% DNase I). Ears were dissected into small pieces. After vigorous shaking of the fragmented tissues, single‐cell suspensions were collected through 70 μm cell strainers (Greiner, Frickenhausen, Germany). Lungs were dissected into small pieces in 3 mL of RPMI 1640 medium and then incubated with 3 mL of Liberase (Roche, Mannheim, Germany) at a final concentration of 2 μg/mL and DNase I (Roche, Mannheim, Germany) at a final concentration of 25 units/mL at 37°C for 30 min. The reaction was stopped by adding 1 mL of heat‐inactivated FCS (Bio&Sell, Nürnberg, Germany) and then the cells were dispersed and filtered, followed by lysing in 1 mL of ACK lysis buffer for 3 min.

### 2.5 Neutrophil Isolation

Neutrophils were isolated by negative selection according to the MojoSort Mouse Neutrophil Isolation Kit (BioLegend, Koblenz, Germany). The biotin antibody cocktail consists of the following antibodies: anti‐mouse CD4, anti‐mouse CD5, anti‐mouse/human CD45R/B220, anti‐mouse CD11c, anti‐mouse CX3CR1, anti‐mouse CD244.2, anti‐mouse F4/80, anti‐mouse CD117 (c‐kit) and anti‐mouse TER‐119/Erythroid Cells. Briefly, BM single‐cell suspensions were prepared without lysis of erythrocytes. Then the cells were resuspended in PBS and incubated on ice for 30 min. After being filtered, the cells were resuspended in MojoSort Buffer (Biolegend, Koblenz, Germany) and then incubated with 10 μL of the Biotin‐Antibody Cocktail, followed by incubation with 10 μL of Streptavidin Nanobeads on ice for 15 min according to the manufacturer's protocol. After being washed and resuspended, the cells were isolated by a magnetic field.

### 2.6 Cell Sorting

BM single‐cell suspensions were stained with PE/Cyanine7‐conjugated anti‐Ly6G and APC‐conjugated anti‐CD11b for 30 min at 4°C (all antibodies from Biolegend, Koblenz, Germany). After washing with ice‐cold PBS, the cell pellets were resuspended in PBS and sorted based upon expression of CD11b and Ly6G by fluorescence‐activated cell sorting (FACS Melody, Becton Dickinson, Heidelberg, Germany). The purity of sorted neutrophils was approximately 97%–98%.

### 2.7 Neutrophil Activation

Isolated neutrophils from BM were activated by 250 ng/mL PMA, 1 μM *N*‐formyl‐methionyl‐leucyl‐phenylalanine (fMLP), (both from Sigma, Taufkirchen, Germany) 50 ng/mL CXCL1, or 50 ng/mL CXCL2 (R&D Systems, Wiesbaden, Germany) in different experiments. In the activation experiments, neutrophils were incubated with fMLP or PMA for 18 h, then stained with anti‐CD45, anti‐CD11b, anti‐Ly6G, anti‐CD62L, anti‐DEC205, and 7‐Aminoactinomycin D (7‐AAD) (all from Biolegend, Koblenz, Germany). In the transmigration experiment, fMLP, CXCL1 and CXCL2 were used as neutrophil chemoattractants. Isolated neutrophils were incubated with them respectively for 2 h and then stained with the surface markers mentioned above.

### 2.8 Sorted‐Neutrophils Intravenous Injection

BM neutrophils from LysM‐eGFP transgenic mice were sorted and washed with PBS twice. Sorted neutrophils were resuspended at a concentration of 5 × 10^6^ cells/mL in PBS. Then, the tail skins of WT mice were disinfected with 70% ethanol, and then 1 × 10^6^ neutrophils in 200 μL of PBS were injected intravenously through the tail vein. All mice were used in the CHS reaction experiment.

### 2.9 Neutrophil Transmigration

Neutrophil transmigration analysis was performed by using a 3 μm pore transwell system (Greiner, Frickenhausen, Germany). Two millilitres of complete medium with 1 μM fMLP or 50 ng/mL CXCL1 or 50 ng/mL CXCL2 was placed in the wells of a six‐well plate. 2 × 10^6^ isolated neutrophils in 1 mL complete medium were added on the top of the transwell inserts and incubated at 37°C for 2 h. After 2 h, the transwell inserts were taken out gently. The medium from the top of the inserts and the bottom wells were both transferred into a new 50 mL tube, then washed with PBS. Subsequently, neutrophils were stained with anti‐CD45, anti‐CD11b, anti‐Ly6G, anti‐DEC205 and 7‐AAD (Biolegend, Koblenz, Germany). To measure the total numbers of transmigrated neutrophils and remaining neutrophils, all cells from each sample were analysed under flow cytometry.

### 2.10 Cytokine Enzyme‐Linked Immunosorbent Assay (ELISA)

Concentrations of CXCL1 in cell‐free peritoneal exudate supernatants and serum were quantified by ELISA according to the manufacturer's protocol of DuoSet ELISA Mouse CXCL1/KC (R&D Systems, Bio‐techne, Wiesbaden, Germany). In brief, 96‐well plates were coated with capture antibody, blocked, and subsequently, serum, peritoneal exudate samples and standards were added into wells. After 2 h of incubation, plates were washed and incubated with secondary reagents and streptavidin‐HRP substrate solution. The optical density was determined using a microplate reader at 450 nm.

### 2.11 In Vitro Neutrophil Maturation

Isolated neutrophils from BM were suspended in complete medium at a concentration of 1 × 10^6^ cells/mL. 100 μL aliquots were seeded in 96‐well plates and cells were cultivated with or without 50 ng/mL granulocyte‐colony stimulating factor (G‐CSF; Bio‐techne, Wiesbaden, Germany) at 37°C. Neutrophils were harvested at Days 1 and 2, and were compared to freshly isolated neutrophils (regard as Day 0). For analysis, cells were stained with anti‐CD45, anti‐CD11b, anti‐Ly6G, anti‐DEC205, anti‐CXCR2, and 7‐AAD (Biolegend, Koblenz, Germany).

### 2.12 Flow Cytometry

**FIGURE 1:**
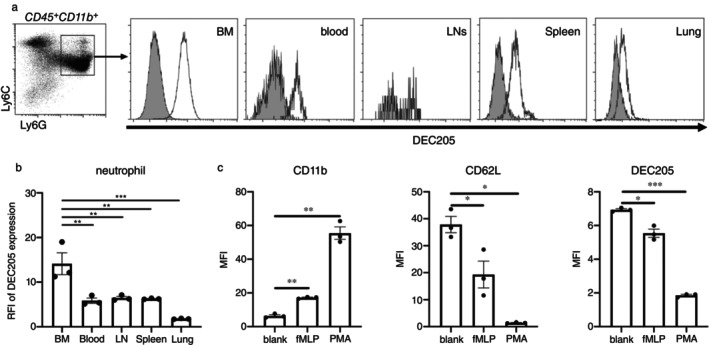
DEC205 is expressed by neutrophils in various tissues and is downregulated upon activation. (a) Representative histograms of DEC205 expression by neutrophils in BM, peripheral blood, LNs, spleens and lungs. The background staining was determined with homozygous B6.129P‐Ly75^tm1Mnz/J^ mice lacking DEC205 (grey). (b) Comparison of DEC205 expression on neutrophils from different tissues. Data were shown as RFI of DEC205. (c) Expressions of CD11b, CD62L and DEC205 on neutrophils activated by fMLP or PMA. Isolated BM‐neutrophils from WT mice were cultivated with fMLP or PMA for 18 h, and marker expression as indicated was analysed by FACS. Data are presented as means ± SEM. Each dot represents one independent experiment. *p < 0.05, **p < 0.01 and ***p < 0.001 (one‐way ANOVA).

To analyse DEC205 expression on neutrophils, single‐cell suspensions were stained with 7‐AAD, anti‐CD45, anti‐CD11b, anti‐Ly6C, anti‐Ly6G, anti‐CD62L and anti‐DEC205 (Biolegend, Koblenz, Germany). Neutrophils were defined as CD45^+^CD11b^+^Ly6C^+^Ly6G^+^ cells (as indicated in Figure 1a). For the other experiments (experiments with two murine inflammatory models, neutrophil in vitro activation analysis, transmigration experiments and maturation experiments), neutrophils were defined as CD45^+^CD11b^+^Ly6G^+^ cells. For the maturation and transmigration experiment, isolated neutrophils were stained with the antibodies mentioned above, as well as anti‐CXCR2. For the identification of neutrophil subsets in BM, the gating strategy from Maximilien Evrard was adopted. BM cells were stained with anti‐CD45, anti‐NK1.1, anti‐B220, anti‐CD115, anti‐Siglec‐F, anti‐c‐KIT, anti‐CD11b, anti‐Ly6G, anti‐CXCR4 and anti‐CXCR2 antibodies (all from Biolegend, Koblenz, Germany). The detailed gating strategy is described in the corresponding paragraph of the results. To determine the expression levels of various adhesion molecules on neutrophils, BM samples were stained with anti‐LFA‐1, anti‐CD11b, anti‐CD62l, anti‐CD29, anti‐CD49d, anti‐CD49b and anti‐CD31, as well as antibodies for neutrophil cell markers (CD45 and Ly6G).

### 2.13 Neutrophil Adhesion

96‐well plates were cultured with 20 μg/mL collagen from rat tail tendon (Roche), human plasma fibronectin (Sigma, Schnelldorf, Germany) and mouse laminin (Roche, Mannheim, Germany) overnight at 4°C, followed by washing with PBS. Then they were blocked with 0.5% BSA in PBS for 1 h at 37°C and washed with PBS. One hundred microliters of isolated neutrophils at the concentration of 1 × 10^6^/mL were added per well in triplicate, and then incubated for 1 h at 37°C. Non‐adherent cells were removed by one gentle wash with 100 μL PBS. Adherent cells were fixed by 100 μL of 4% paraformaldehyde for 15 min. Then adherent cells were stained with 1:10 diluted DAPI (Thermo Fisher, Dreieich, Germany). The numbers of adherent cells were recorded per field of view under a ×10 objective (Evos7; Thermo Fisher, Dreieich, Germany).

### 2.14 Statistics

Statistical analyses of data were performed by using GraphPad Prism 9 (Witzenhausen, Germany) and data in the figures were presented as mean ± SEM. For comparisons within two groups, unpaired Student's *t* test was used to generate *p* values. Analyses of significant differences among more than two groups were assessed by one‐way analysis of variance (ANOVA), two‐way ANOVA or multiple *t* test. GraphPad Prism 9.0 and Kaluza software 2.1 (Beckman Coulter, Krefeld, Germany) were used to generate diagrams and figures. Different levels of significance are statistically considered as *p < 0.05, **p < 0.01, ***p < 0.001 and ****p < 0.0001, whereas non‐significant *p*‐values are unlabelled.

## 3 Results

### 3.1 DEC205 Expression by Murine Neutrophils and Its Regulation by Activation

To investigate the expression of DEC205 on neutrophils, various neutrophil subpopulations derived from BM, peripheral blood, LNs, spleens, and lungs were analysed by flow cytometry. Neutrophils were defined by being CD3^−^, CD19^−^, CD45^+^, CD11b^+^, Ly6C^+^ and Ly6G^+^, and expression of DEC205 in WT mice was analysed in comparison to DEC205^−/−^ mice. The results indicate that neutrophils isolated from the respective organs express DEC205 (Figure 1a). When comparing the relative fluorescence intensities (RFIs) of DEC205 expression in different organs (Figure 1b), the most prominent expression was recorded in BM‐derived neutrophils, followed by neutrophils residing in blood, LNs, spleens and lungs. To assess whether activation of neutrophils alters DEC205 expression, cells were isolated from WT mice, followed by cell culture with fMLP or PMA, two potent activators of neutrophils. After overnight cultivation, WT neutrophils upregulated CD11b expression and downregulated CD62L expression, as compared to unstimulated (blank) control groups (Figure 1c). This indicates activation of the neutrophils. In parallel, this activation reduced expression of DEC205 (Figure 1c).

### 3.2 Reduction of DEC205 Expression by Activated Neutrophils in Peripheral Tissues

**FIGURE 2:**
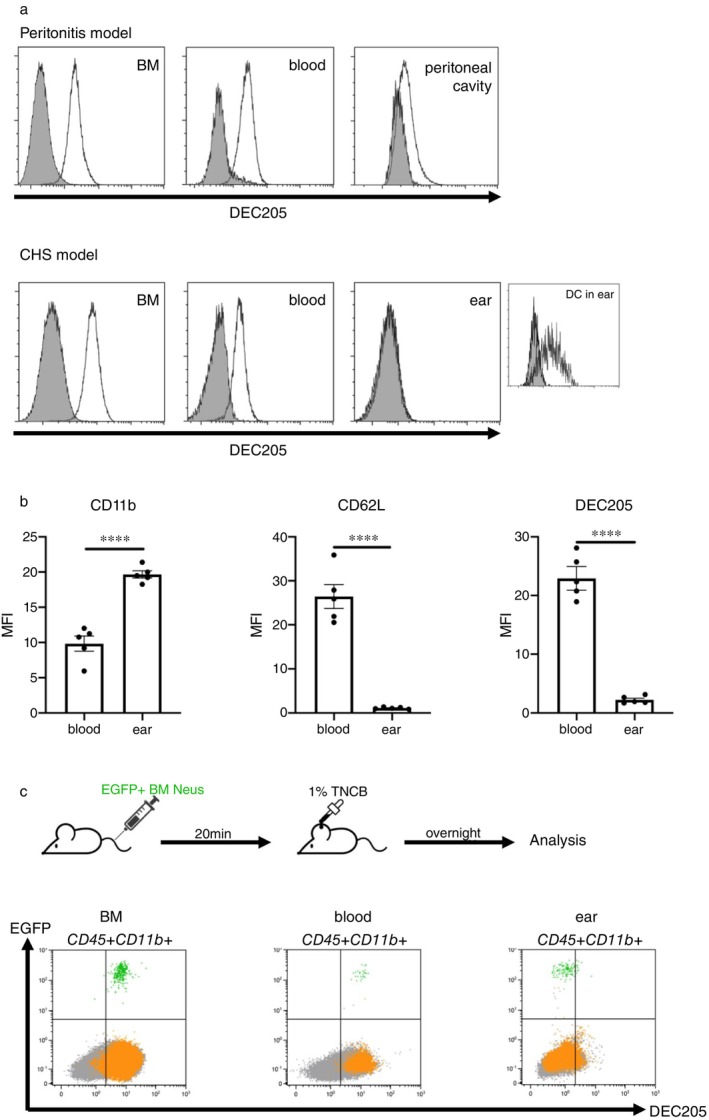
Expression of DEC205 by neutrophils is downregulated during activation and migration to peripheral tissues. (a) Representative histograms of DEC205 expressions by neutrophils in different tissues of mice with experimentally generated peritonitis and CHS. The expression of DEC205 by DC in inflamed ear served as positive control in CHS. The background staining was determined with homozygous B6.129P‐Ly75tm1Mnz/J mice lacking DEC205 (displayed in grey). (b) Comparisons of expressions of CD11b, CD62L and DEC205 by neutrophils in blood and inflamed ears. Data are shown as means ± SEM of MFI. (c) Intravenous injection of sorted EGFP^+^DEC205^+^ BM neutrophils (Neus) into syngeneic hosts. BM‐neutrophils were sorted from B6/EGFP transgenic mice and injected intravenously into WT mice. The recipient mice were sensitised on ears with 1% TNCB and after overnight BM, blood and ears were collected and analysed by flow cytometry. The results shown are representative of three experiments with three animals each. The green population (EGFP^+^Ly6C^+^Ly6G^+^) depicts injected‐neutrophils from B6/EGFP transgenic mice and the yellow population (EGFP^−^Ly6C^+^Ly6G^+^) displays recipient's autologous neutrophils. ****p < 0.0001 (unpaired Student's *t* test).

In vivo, neutrophils become activated upon exposure to signals derived from inflammation, infection or tissue damage. To investigate changes in DEC205 expression under these conditions, we analysed neutrophils in two murine inflammatory models: TNCB‐induced CHS and thioglycolate‐induced peritonitis. DEC205 expression was examined in the BM (source of circulating neutrophils), blood (where neutrophils are mobilised), and inflamed tissues (where neutrophils are recruited for immune responses). Figure 2a shows a significant decrease in DEC205 expression in neutrophils across these compartments in both the CHS and peritonitis models.

In the CHS model, an inverse correlation was observed between DEC205 expression and neutrophil activation status; specifically, DEC205 expression decreased as activation occurred, as indicated by the upregulation of CD11b and downregulation of CD62L (Figure 2b).

In line with observations of Ballesteros et al. [21], in uninflamed skin only very limited numbers of neutrophils (0.5%–1.3% of all CD45^+^ ear cells; data not shown) were detectable. These in vivo data are in line with our in vitro results, which also showed downregulation of DEC205 expression following neutrophil activation.

Since neutrophils recruited from blood to tissues appear to lose DEC205 expression, we next transferred DEC205+ BM‐derived neutrophils from B6 EGFP^+^ transgenic mice into syngeneic hosts via tail‐vein injection, followed by sensitisation with 1% TNCB. After 18 h, the distribution of donor neutrophils in tissues was analysed. EGFP^+^DEC205^+^ BM‐derived neutrophils were identified in the BM, blood, and ears of sensitised WT recipient mice. In the BM and blood, these neutrophils maintained DEC205 expression, whereas they exhibited a DEC205‐negative phenotype in the inflamed ears (Figure 2c).

Altogether, our data demonstrate that neutrophils transmigrating from blood to inflammatory tissues become fully activated and downregulate DEC205 expression.

### 3.3 Expression of Various Adhesion Molecules by Neutrophils and Its Impaired Adhesion Capacity to Laminin by Lacking DEC205

**FIGURE 3:**
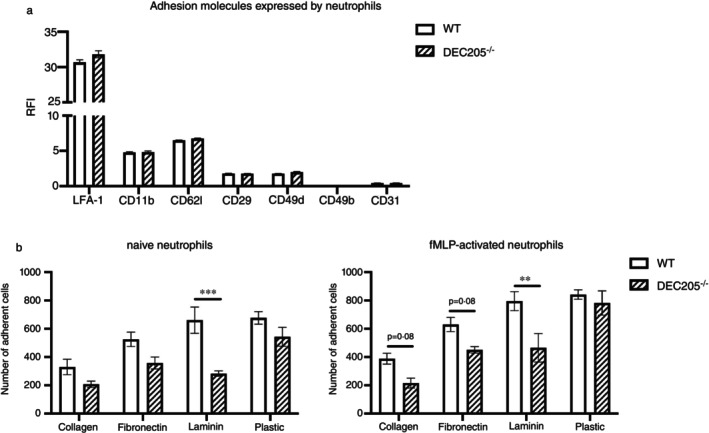
Lack of DEC205 impaired the adhesive capability of neutrophils to laminin. (a) Expressions of LFA‐1, CD11b, CD62L, CD29, CD49d, CD49b and CD31 by BM neutrophils from WT and DEC205^−/−^ mice. The expressions of adhesion molecules were analysed by FACS. Data are shown in the form of RFI, using fluorescence minus one as control. (b) Comparisons of the adhesive capability of naïve or fMLP‐activated neutrophils from WT and DEC205^−/−^ mice. Half of the isolated BM‐neutrophils from WT and DEC205^−/−^ mice were seeded in plates coated with different ECM proteins, and the other half of the neutrophils were cultured with 1 μM fMLP additionally. Then non‐adherent cells were removed after 1 h of cultivation, and adherent cells were fixed by 4% paraformaldehyde. Data are shown in the form of the mean ± SEM of adherent cell numbers from three independent experiments. **p < 0.01 and ***p < 0.001 (multiple *t* test).

The recruitment cascade of neutrophils has been well investigated and involves a commonly recognised pathway: tethering, rolling, adhesion, crawling and transmigration. During this process, various adhesion molecules are involved. Since we have observed that neutrophils downregulate DEC205 after migration from blood to peripheral tissues, DEC205 may either be involved in regulating the adhesion of neutrophils to a so far unknown ligand or at least DEC205 may serve as an indicator for rather immature neutrophils with a capacity to migrate to peripheral tissues. To analyse a potential correlation of DEC205 expression with the expression of defined adhesion molecules, flow cytometric analysis of WT and DEC205^−/−^ neutrophils was performed. LFA‐1, CD11b (which forms MAC‐1 with CD18), CD49d/CD29 (VLA‐4), CD49b/CD29 (VLA‐2), CD62L and CD31 were expressed in WT and DEC205^−/−^ neutrophils at similar levels (Figure 3a), indicating that lack of DEC205 does not alter the expression profile of these adhesion molecules in neutrophils. Next, isolated neutrophils were seeded onto plates coated with different ECM proteins to assess their ability to adhere. As shown in Figure 3b, fewer DEC205^−/−^ neutrophils adhered to laminin‐coated plates compared with WT neutrophils. As expected, the number of adherent neutrophils was up‐regulated among all groups after activation by fMLP. However, DEC205^−/−^ neutrophils still adhered less than WT neutrophils to laminin‐treated surfaces. These data, showing that the absence of DEC205 reduced the adhesion of neutrophils to laminin without altering defined laminin‐binding molecules (VLA2), indicate that DEC205 may mediate adhesion to laminin.

### 3.4 Decreased Migration of DEC205 ^−/−^ Neutrophils in Boyden Chambers

**FIGURE 4:**
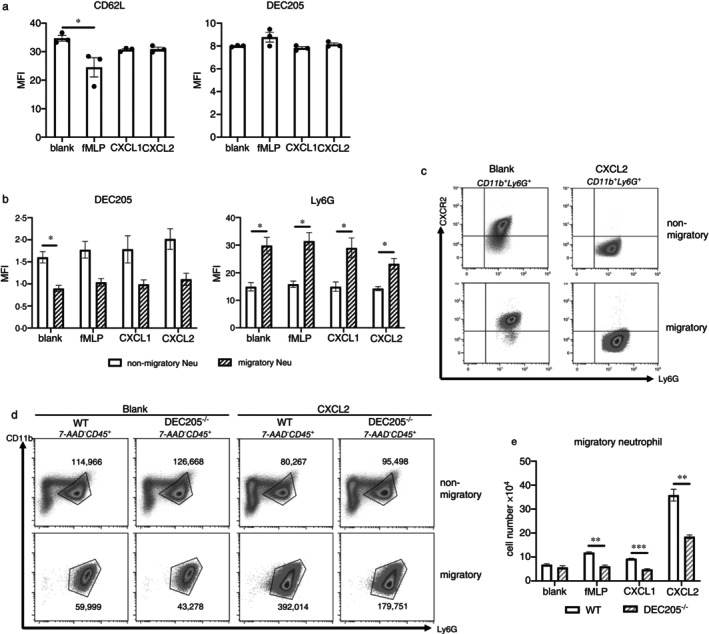
DEC205^−/−^ neutrophils display impaired migration in vitro. (a) Isolated BM‐neutrophils were cultured with fMLP, CXCL1 or CXCL2 overnight, and expression of CD62L and DEC205 was compared among all groups. (b–e) Isolated BM‐neutrophil migration through 3.0‐μm transwell inserts towards chemotactic agents was assessed. (b) Change of DEC205 and Ly6G expression in neutrophils before and after transmigration. (c) Representative histograms of Ly6G and CXCR2 expression by neutrophils before and after transmigration. Shown are the blank control group and the CXCL2 group. (d) Representative histograms of WT and DEC205^−/−^ neutrophils before and after transmigration in blank controls and the CXCL2 group. Neutrophils were defined as CD11b^+^Ly6G^+^ population and numbers in histograms indicate migratory and non‐migratory neutrophils. (e) Comparison of the average numbers of migratory WT and DEC205^−/−^ neutrophils. Data are presented as the mean ± SEM of three independent experiments. *p < 0.05, **p < 0.01, ***p < 0.001 (one‐way ANOVA for a, multiple *t* test for b and d).

As our data from the murine inflammatory models indicate involvement of DEC205 in neutrophil adhesion and migration in vivo, we further investigated the migratory behaviour of DEC205^+^ BM‐derived neutrophils in Boyden chamber experiments in vitro. First, we cultured isolated neutrophils with different chemoattractants, that is, fMLP, CXCL1 and CXCL2, in order to define whether these chemicals themselves can already regulate DEC205 expression in neutrophils. The results show that expression of DEC205 remained stable in control‐ and chemoattractant‐treated groups (Figure 4a). However, CD62L expression was slightly (but not statistically significant) down‐regulated in all chemoattractant groups, indicating partial activation of neutrophils. Altogether, these data indicate that the respective cytokines rather act as chemoattractants, but not as activators of neutrophils.

Next, isolated neutrophils were cultivated for 2 h in Boyden chambers with the respective chemoattractant added to the lower chamber. Non‐migrated neutrophils in the upper chamber and migratory neutrophils in the lower chamber were collected and assessed for expression of DEC205, Ly6G and other neutrophil surface markers by flow cytometry. The results show that migratory neutrophils displayed increased expression of Ly6G and a decreased expression of DEC205, as compared with non‐migratory neutrophils in all groups (Figure 4b). As CXCL2 is the prototypic neutrophil attractant in vivo, the effects on migration were analysed in more detail (Figure 4c). The receptor for CXCL2, CXCR2, was downregulated by incubation of the cells with CXCL2. Migratory neutrophils expressed higher Ly6G and almost all of them were CXCR2‐positive in the blank control group. Because CXCR2 and Ly6G are neutrophil maturation markers, this result indicates that preferentially mature neutrophils migrated through the membranes, whereby a decrease in DEC205 expression occurred.

To assess whether the expression of DEC205 is mandatory to facilitate migration, WT and DEC205^−/−^ neutrophils were subjected to Boyden chamber migration experiments. Here, representative histograms of numbers of migratory neutrophils from WT and DEC205^−/−^ mice between blank control group and CXCL2 group are shown in Figure 4d. CXCL2 attracted twofold more WT neutrophils to migrate compared to DEC205^−/−^ neutrophils, while the numbers of migratory neutrophils between WT and DEC205^−/−^ were at the same level in the control group. It also shows that there were more non‐migratory DEC205^−/−^ neutrophils compared to WT neutrophils in the CXCL2 group. However, the number of non‐migratory neutrophils did not correspond to that of migratory neutrophils, which could be caused by not including the cells attached to the membrane. In a more comprehensive approach, different chemoattractants were tested in Boyden chambers (Figure 4e). Again, the migration of DEC205^−/−^ neutrophils was significantly reduced as compared to WT neutrophils in all groups (fMLP, CXCL1, CXCL2) investigated. Thus, these data support our hypothesis that DEC205 is critically involved in neutrophil migration.

### 3.5 Impaired Migration of Neutrophils From DEC205 ^−/−^ Mice In Vivo

To support our hypothesis that DEC205 is necessary for the migration of neutrophils in vivo, acute peritonitis was induced by intraperitoneal injection of thioglycolate into WT and DEC205^−/−^ mice, followed by the analysis of populations of neutrophils in the BM, blood, and PECs at different time points.

**FIGURE 5:**
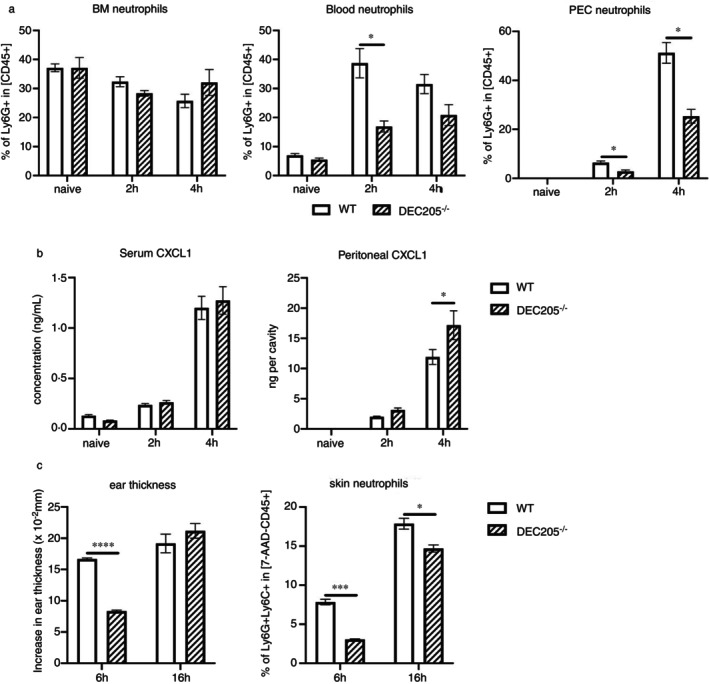
DEC205^−/−^ neutrophils display impaired migration to blood and peritoneal cavity in vivo. (a) Frequencies of neutrophils in different tissues of WT and DEC205^−/−^ mice with experimentally generated peritonitis. BM, peripheral blood and PECs were collected from naïve mice and thioglycolate‐injected mice at 2 and 4 h, and percentages of neutrophils were analysed by flow cytometry. Mean values ± SEM; *n* = 3; * indicates *p* < 0.05 (multiple *t* test). (b) Blood and peritoneal exudates from peritonitis‐induced WT and DEC205^−/−^ mice were collected at different time points. CXCL1 content was analysed by ELISA. (c) Increase in ear thickness, as compared to pre‐challenge, and frequencies of neutrophils in ears (6 and 16 h after challenge) was analysed in WT and DEC205^−/−^ mice. Data are shown as mean ± SEM. *p < 0.05, ***p < 0.001, ****p < 0.0001 (multiple *t* test).

Figure 5a shows that DEC205^−/−^ mice harboured significantly lower numbers of neutrophils in blood 2–4 h after thioglycolate injection. In detail, at 2 h less neutrophils (16.89% ± 3.34%) were recruited to the blood in DEC205^−/−^ mice compared to WT mice (38.71% ± 8.79%). At 4 h, the percentage of blood neutrophils decreased in WT mice, while it increased even further in DEC205^−/−^ mice, indicating that neutrophil recruitment to the blood had already peaked in WT mice but not yet in DEC205^−/−^ mice. However, in the BM, the percentages of neutrophils at all time points were not different between both mouse lines (Figure 5a).

When analysing PEC samples, differences were apparent again. That is, DEC205^−/−^ mice harboured fewer neutrophils in their peritoneal cavity than WT controls and only negligible percentages of neutrophils were detected in naïve mice from both groups (Figure 5a). Other cell types such as monocytes, B‐ and T cells were not detected in the peritoneal cavity; therefore, the increase in neutrophils reflects the total number of exudate cells in DEC205^−/−^ animals and WT littermates.

Additionally, the concentration of CXCL1, which is the major chemokine responsible for recruiting neutrophils to peripheral tissues, was tested in blood and peritoneal cavity. As depicted in Figure 5b, induction of peritonitis induced CXCL1 in WT and DEC205^−/−^ mice to a similar extent. Therefore, a failure of production of neutrophilic chemokines by DEC205^−/−^ mice does not account for the reduced infiltration; rather, these data indicate that DEC205^‐^ neutrophils are impaired in migrating from BM into blood and further into peripheral tissues. In a further model of inflammation, mice were subjected to TNCB‐induced CHS reactions (Figure 5c). The analysis revealed that 6 h after challenge in a TNCB model, significantly fewer neutrophils were detected in ear skin in DEC205^−/−^ mice as compared to WT littermates. This corresponds to the reduced ear swelling reaction obtained in these mice. However, 16 h after challenge, the ear swelling as well as the numbers of the skin infiltrating neutrophils levelled out between WT and DEC205^−/−^ mice. Thus, while not a complete blockage of skin migrating neutrophils could be observed, these data support the idea that DEC205 expressed by neutrophils may play a role in adherence and positioning of neutrophils during inflammatory conditions in peripheral tissues.

### 3.6 Altered Expression Levels of DEC205 During Neutrophil Maturation and the Retention of DEC205 ^−/−^ Neutrophils in the BM During Inflammation

**FIGURE 6:**
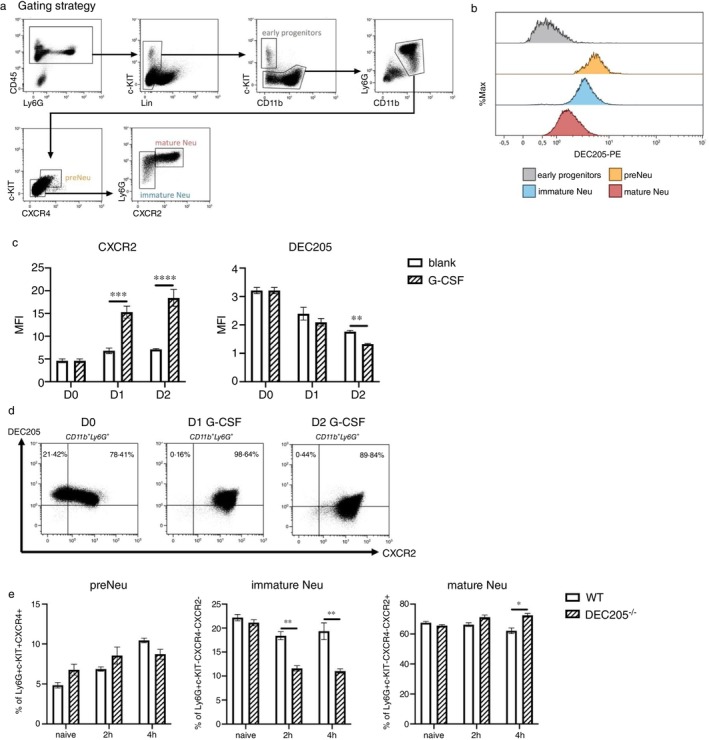
DEC205 expression is decreased during neutrophil maturation and DEC205^−/−^ neutrophils are retained in BM during inflammation. (a) Gating strategy of distinguishing BM‐neutrophil subsets. (b) Representative histograms of DEC205 expression level among different BM‐neutrophil subsets. (c) Change of DEC205 and CXCR2 expression on neutrophils after culture with G‐CSF. Isolated BM neutrophils were cultivated with or without 50 ng/mL G‐CSF for 2 days. CXCR2 and DEC205 expression were assessed every day. The results are representative of three independent experiments. (d) Representative plots of DEC205 and CXCR2 expressions on neutrophils in different groups. (e) Comparison of BM neutrophil subsets' percentages between WT and DEC205^−/−^ mice with experimentally generated peritonitis. The mouse model was induced as previously described. Data are expressed as frequencies of preNeu, immature Neu and mature Neu in the whole BM neutrophil population and are representative of four independent experiments. *p < 0.05, **p < 0.01, ***p < 0.001 and ****p < 0.0001 (two‐way ANOVA for c and multiple *t* test for e).

We observed that the expression of DEC205 by BM neutrophils is variable and CD45^+^CD11b^+^Ly6C^+^Ly6G^+^ neutrophils in the BM comprise not only mature neutrophils, but also immature neutrophils as well as several neutrophil progenitors. Therefore, we set out to analyse the frequency and development of neutrophil subsets in the BM of WT in comparison to DEC205^−/−^ mice. Different subsets of neutrophils in BM were analysed according to a gating strategy adopted from Evrard [22], as shown in Figure 6a. At first, CD45^+^ cells were gated, followed by excluding NK1.1, B220, CD115 and Siglec‐F positive cells. Within this ‘lineage‐negative gate’ the c‐KIT‐positive population was termed early progenitors. In the c‐KIT^−^CD11b^+^Ly6G^+^ gate, a pre‐neutrophil precursor (preNeu) population was defined by the high expression levels of c‐KIT and CXCR4, while mature neutrophils (mature Neu) and immature neutrophils (immature Neu) were defined by the different expression levels of CXCR2.

We found that DEC205 was not expressed by early progenitors; however, DEC205 started to be clearly expressed by preNeu and reduced its expression in immature and mature neutrophils (Figure 6b). When inducing maturation of BM‐derived neutrophils by incubation with G‐CSF in vitro, DEC205 expression ceased as they became more mature (as indicated by CXCR2 expression). Although a decrease in DEC205 expression over time on neutrophils was also observed in the blank control group, neutrophils cultured with G‐CSF exhibited a more pronounced reduction in DEC205 expression, particularly, evident on the second day of cultivation (Figure 6c). Additionally, as shown in Figure 5d, the proportion of immature (CXCR2^−^DEC205^high^) neutrophils decreased within the culture over time. Thus, these data in the BM corroborate our notion that neutrophils in the peripheral blood continue to downregulate DEC205 expression upon maturation.

Moreover, the maturation of preNeu, immature Neu and mature Neu populations in the BM of mice with thioglycolate‐induced peritonitis was examined. Four hours after induction of inflammation, DEC205^−/−^ mice had higher frequencies of mature and lower numbers of immature neutrophils as compared to WT mice, respectively. Together with our data showing decreased numbers of neutrophils in the blood of DEC205^−/−^ mice during peritonitis, these data indicate that DEC205^−/−^ neutrophils may have a defect in leaving the BM to enlarge the pool of blood‐circulating neutrophils, and already at the immature stage the replenishment of neutrophils taking place in WT BM seems to be delayed in DEC205^−/−^ mice, as here only lower numbers are recorded.

In aggregate, these data show that DEC205 expression by neutrophils acts as a marker for functionally immature neutrophils in the blood and may guide their migration from BM to peripheral tissues by acting as an adhesion molecule.

## 4 Discussion

Under homeostatic conditions, the expression of DEC205 by neutrophils, B cells and T cells was detected, while monocytes lacked DEC205 expression. Neutrophils expressed the highest levels of DEC205 in the BM, but this decreased once neutrophils entered the blood, LNs, spleen, and lungs. These findings are consistent with a study by Inaba et al. on the tissue distribution of DEC205 [14]. Although neutrophils, B cells and T cells can express DEC205, the levels reached by CD8^+^ conventional DCs were 6‐ to 15‐fold higher. Therefore, DEC205 was thought to be exclusively expressed by DCs among all leukocytes.

In DCs, DEC205 has been assigned the function of a receptor for absorptive endocytosis, and neutrophils are indeed able to stimulate T cells by presentation of antigens [23, 24, 25], in mice as well as in humans [12, 26].

However, while DCs are termed professional antigen‐presenting cells, neutrophils are considered as ‘atypical’ antigen‐presenting cells [27], as they need certain stimuli (i.e., chemo‐ and/or cytokines) to acquire their antigen‐presenting function [28]. Therefore, DEC205 may contribute to antigen presentation by neutrophils under circumstances that have not been clarified yet. Alternatively, in non‐professional antigen‐presenting cells, such as neutrophils, DEC205 may have acquired functions, beyond acting as an antigen uptake receptor. As a potential novel function for DEC205, we report here its involvement in mechanisms of cellular adhesion and migration.

So far, DEC205 has not been characterised as being involved in mediating cell adhesion. However, it contains 10 so‐called carbohydrate recognition domains (CRDs) [29, 30]. These domains specifically recognise and bind to carbohydrate structures, typically those on glycoproteins or glycolipids [31, 32]. The main ligands for CRDs are therefore carbohydrates, and the binding is often calcium‐dependent, which is a hallmark of the C‐type lectin family. These properties enable C‐type lectins to act as adhesion molecules for pathogens. For instance, the dendritic cell‐specific ICAM‐3‐grabbing non‐integrin (DC‐SIGN, CD209) binds to mannose‐rich glycans on pathogens and is responsible for binding HIV [33, 34]. Langerin (CD207), another C‐type lectin receptor expressed by DCs, is able to bind and endocytose β‐glucan‐exhibiting pathogens, such as certain viruses, yeast and Candida [35].

So far, the binding of CRD domains to carbohydrates has primarily been investigated in the context of the uptake of pathogenic antigens. However, beyond pathogens, extensive carbohydrate structures can also be expressed by cells and may serve as recognition structures for adhesion molecules of the selectin type, that is, Type I transmembrane proteins with a single C‐type lectin domain, such as CD62L, CD62E and CD62P [36]. These selectins can bind a diverse group of oligosaccharides [37], all of which play an important role in leukocyte adhesion in different tissues. As for the mannose receptor (MMR), which is the closest relative to DEC205, decreased adhesion of lymphocytes to lymphatic vessels from MMR‐deficient mice [38] and increased random cell migration of macrophages [39] have been reported. Therefore, based on these findings, it is conceivable that DEC205 may engage carbohydrate ligands during the process of endothelial transmigration, potentially influencing neutrophil migration.

In addition to CRD domains, the extracellular part of the DEC205 receptor contains a common extracellular arrangement: an amino‐terminal cysteine‐rich domain followed by a fibronectin Type II domain (FNII) [29]. This FNII sequence is shared by the MMR, the endo180 receptor and the phospholipase A2 receptor. The FNII sequence in DEC205 may contribute to cell migration and adhesion, as it has been shown that a truncated Endo180 protein lacking the FNII domain, expressed by embryonic fibroblasts, results in cells with a defect in collagen binding and internalisation and an impaired migratory phenotype [40]. Likewise, a truncated version of the phospholipase A2 receptor, which is missing the FNII domain, causes a loss of binding to collagen and a reduction in collagen‐dependent migration of a human HEK cell line [41]. Of note, the MMR, which is the closest relative to DEC205, can not only bind to carbohydrates but also to ECM proteins, such as collagen, which enables it to play a role in mediating cell‐matrix adhesion [42]. Thus, our data showing decreased adhesion of DEC205^−/−^ neutrophils to ECM proteins and reduced migration to peripheral tissues in vivo are consistent with our hypothesis that DEC205 is directly or indirectly involved in regulating adhesion in neutrophils.

Regarding direct involvement, the structure of DEC205 is well characterised and supports our hypothesis that it may act as an adhesion molecule. However, no specific ligands involved in adhesion have been identified for DEC205 to date. Therefore, a definitive characterisation of DEC205 as an adhesion molecule in neutrophils may be premature. Nevertheless, DEC205 appears to serve an important function as a molecular marker for relatively immature neutrophils en route to peripheral tissues.

Indeed, in vivo, a complex network of soluble (e.g., chemokine gradients) and solid (e.g., ECM proteins, epithelial cells) cues guides maturation and migration of neutrophils from the BM to sites of infection, with the chemokines CXCL1 and CXCL2 being the most potent neutrophil attractants in vivo [43, 44]. During this maturation process, neutrophils gradually downregulate the surface expression of DEC205 before they reach their final destination. This downregulation of DEC205 is even detectable within the BM, where the expression of DEC205 gradually decreases from the stage of pre‐neutrophils to immature and, finally, mature neutrophils. This may further imply a relationship between DEC205 expression and functional differentiation. That is, early, BM‐born neutrophils may require DEC205 expression to sequentially mature and pass through the BM parenchyma towards the BM sinusoids [45, 46], eventually entering the bloodstream. In support of this hypothesis, a stalled release of neutrophils from the BM was observed in DEC205^−/−^ animals. However, once the neutrophils have reached their final destination and are functionally mature, DEC205 is no longer needed for further migration and peripheral targeting.

In summary, this study reports a novel function for the DEC205 receptor in neutrophils. Beyond its well‐established properties as an antigen uptake receptor in professional antigen‐presenting cells, it acts as a marker molecule in neutrophils that is downregulated during functional differentiation. Moreover, its possible involvement in adhesion and migration processes may enable neutrophils to ‘sample’ their substrate during migration through different tissue compartments. As neutrophils are indeed able to take up and present antigens after appropriate activation [12, 47, 48], the DEC205 receptor may provide tissue cues to support the induction of adaptive immunity by antigen presentation and mediating the functional maturation of neutrophils [49].

## Author Contributions

S.J. designed and performed experiments, wrote the initial draft of the manuscript. X.L. designed and analysed experiments. S.S. performed experiments. A.E. wrote the paper. K.M. designed experiments and wrote the manuscript.

## Ethics Statement

This study and its experimental procedures were approved by the State of Baden Württemberg. All animal housing and experiments were conducted in accordance with institutional guidelines for the care and use of laboratory animals.

## Conflicts of Interest

The authors declare no conflicts of interest.

## Data Availability

The data that support the findings of this study are available on request from the corresponding author. The data are not publicly available due to privacy or ethical restrictions.
